# Development and Relative Validity of a Semiquantitative Food Frequency Questionnaire to Estimate Dietary Intake among a Multi-Ethnic Population in the Malaysian Cohort Project

**DOI:** 10.3390/nu13041163

**Published:** 2021-04-01

**Authors:** Suzana Shahar, Mohd Razif Shahril, Noraidatulakma Abdullah, Boekhtiar Borhanuddin, Mohd Arman Kamaruddin, Nurul Ain Md Yusuf, Andri Dauni, Hanisah Rosli, Nurzetty Sofia Zainuddin, Rahman Jamal

**Affiliations:** 1 Centre for Healthy Aging and Wellness, Faculty of Health Sciences, Universiti Kebangsaan Malaysia, Kuala Lumpur 50300, Malaysia; razifshahril@ukm.edu.my; 2 UKM Medical Molecular Biology Institute, Universiti Kebangsaan Malaysia, Kuala Lumpur 56000, Malaysia; noraidatulakma.abdullah@ppukm.ukm.edu.my (N.A.); boekhtiar@yahoo.com (B.B.); arman@ppukm.ukm.edu.my (M.A.K.); ainmdy@ukm.edu.my (N.A.M.Y.); andri@ppukm.ukm.edu.my (A.D.); 3 Faculty of Allied Health Sciences, Cyberjaya University College of Medical Sciences, Persiaran Bestari, Cyberjaya 63000, Malaysia; hanisahrosli@yahoo.com; 4 School of Health Sciences, Universiti Sains Malaysia, Kota Bharu 16150, Malaysia; zettysofia@usm.my

**Keywords:** food frequency questionnaire, relative validity, dietary intake, The Malaysian Cohort

## Abstract

Measuring dietary intakes in a multi-ethnic and multicultural setting, such as Malaysia, remains a challenge due to its diversity. This study aims to develop and evaluate the relative validity of an interviewer-administered food frequency questionnaire (FFQ) in assessing the habitual dietary exposure of The Malaysian Cohort (TMC) participants. We developed a nutrient database (with 203 items) based on various food consumption tables, and 803 participants were involved in this study. The output of the FFQ was then validated against three-day 24-h dietary recalls (n = 64). We assessed the relative validity and its agreement using various methods, such as Spearman’s correlation, weighed Kappa, intraclass correlation coefficient (ICC), and Bland–Altman analysis. Spearman’s correlation coefficient ranged from 0.24 (vitamin C) to 0.46 (carbohydrate), and almost all nutrients had correlation coefficients above 0.3, except for vitamin C and sodium. Intraclass correlation coefficients ranged from −0.01 (calcium) to 0.59 (carbohydrates), and weighted Kappa exceeded 0.4 for 50% of nutrients. In short, TMC’s FFQ appears to have good relative validity for the assessment of nutrient intake among its participants, as compared to the three-day 24-h dietary recalls. However, estimates for iron, vitamin A, and vitamin C should be interpreted with caution.

## 1. Introduction

Noncommunicable diseases are increasing in prevalence and are now the major causes of morbidity and mortality in Malaysia, a developing country, comparable to that of developed countries. The rising trends are most likely a consequence of the demographic and dietary transition and the globalization of economic processes. Within a span of five years (2011–2015), data from the National Health Morbidity Survey showed that the prevalence of noncommunicable diseases in Malaysia has increased, i.e., diabetes from 15.2% to 17.5%, hypercholesterolemia from 35.1% to 47.7%, and obesity from 15.1% to 17.7% [1]. In addressing the rising trends, The Malaysian Cohort (TMC) project, which was initiated by the government in 2006, took up the challenge to investigate the interaction between environmental factors, including diet, and noncommunicable diseases in this multi-ethnic population through a large prospective study involving 106,527 participants [2]. Measuring dietary intakes of free-living individuals is a tedious task, because of the variability in food preference and diversity of mixed ethnic dishes. Thus, a dietary intake assessment tool suitable for a large-scale epidemiologic study is required.

Food frequency questionnaires (FFQs) have been commonly used as a tool to reflect the accuracy of dietary intake [3]. The major advantage of the FFQ, unlike other methods, such as diet records or recalls, is the ability to assess usual dietary intakes over a longer period of time. Thus, it is often being used in large scale epidemiological study to investigate the relationship between diet and disease. It also appears to be low in cost during both administration and analyzing process [4]. Several major prospective studies, such as the Nurses’ Health Study, UK Women’s Cohort Study, and European Prospective Investigation into Cancer and Nutrition (EPIC) Study, have used FFQs developed specifically for their populations [5,6,7]. This is because the FFQ developed for one population is not applicable or shared with another population, due to the differences in culturally specific food intake patterns. Numerous FFQs have been developed worldwide, since each population would have their list of foods common to their dietary consumption patterns [8]. Currently, available FFQs in Malaysia were developed using small-scale studies involving specific age groups and nutrients and are unable to represent the Malaysian multi-ethnic population, in general [9,10,11,12,13]. Therefore, this paper describes the development of a semiquantitative FFQ to estimate dietary intake among a multi-ethnic population. It will be used in TMC project in identifying diet–disease relationships in relation to noncommunicable diseases.

## 2. Materials and Methods

This study was carried out in two phases. Phase 1 involved data analysis of food intake as assessed, using a combination of a 24 h recall and two diet records of 803 participants in TMC’s sample to develop the FFQ. This phase involves development of a food list and nutrient database. Phase 2 is a validation of the FFQ among a different sample from which the FFQ had been developed. The validation was conducted among 64 TMC samples, which were selected from an urban and a rural area of Malaysia, and intake from the FFQ was compared against a three-day 24-h recall.

### 2.1. Study Sample

**Figure 1:**
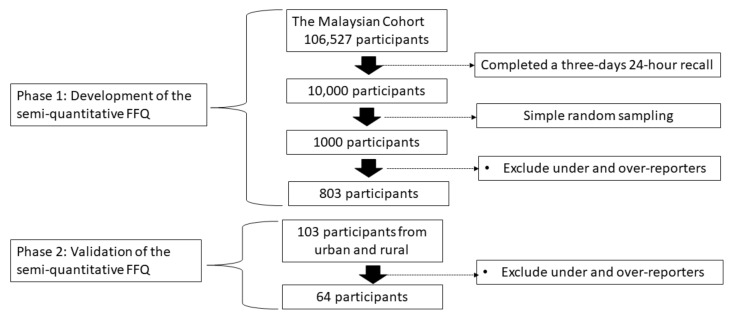
Flowchart on sample selection. FFQ denotes as Food Frequency Questionnaires

In Phase 1, food intake data were collected from a subsample of 10,000 participants purposely selected from the 106,527 participants of The Malaysian Cohort (TMC) project that had returned a three-day 24-h recall, aged 35 to 70 years, who were recruited between April 2006 and September 2012 [2]. From the 10,000 samples, a subsample of 1000 were selected using simple random sampling, and from that, 803 subjects were selected, based on availability of complete data of the 24-h diet recall and the two-day food records after excluding under- and over-reporters, to represent the TMC participants for the development of the semiquantitative FFQ. In phase 2, a validation study was conducted among 103 subjects recruited conveniently from 28 September to 25 November 2018. These subjects were recruited purposively from participants being followed-up in TMC project. The urban subjects were from Kuala Lumpur, while the rural subjects were from an agricultural settlement (Felda Besout) in Perak, a state on the west coast of Malaysia. After data cleaning, only 64 participants were eligible to be included in the validation study. Those with an energy intake of less than 1200 kcal and more than 3000 kcal were considered as under- and over-reporters, respectively, and, thus, excluded from the analysis [14] (Figure 1). The inclusion criteria to TMC project included being a Malaysian citizen, possession of a valid nationality identification card, not suffering from any acute illness at the time of study, and those who gave written informed consent to the study. Those with debilitating illnesses, including cancers, and those who refused consent were excluded. This study was conducted according to the guidelines laid down in the Declaration of Helsinki, and all procedures involving research study participants were approved by the Ethics Committee of Universiti Kebangsaan Malaysia (Project Code: FF-205-2007). Written informed consent was obtained from all subjects/patients prior to study.

### 2.2. Data Collection

The cohort sampling was performed using a mixed approach of cluster and targeted sampling. Details on the sampling were published elsewhere [2]. Various information on demography, medical, and occupational history were collected using questionnaires and interviews. Each cohort participant had their height and weight measured to calculate their body mass index (BMI). Dietary intake was assessed using an interview-administered 24-h recall using multiple pass approach and a nonconsecutive 2-day food record, which also includes one weekend, using a tablet personal computer with touch-screen features. Key pop-up features included a data dictionary, as well as a digital diet album, to assist both the enumerators and the participants to estimate their food portion sizes. Participants were asked about all details, including type of food, cooking methods, estimated portion sizes, and brand of food and beverages consumed for the past 24 h. Each participant was interviewed face-to-face by a trained interviewer, and the session was recorded with consent using the tablet computer recording system as well as an MP3 player. Every interview recording was listened to and audited by an independent enumerator. The errors were coded and rectified accordingly as a quality control measure.

### 2.3. Development of the Food List

All food items and mixed dishes obtained using the 24-h recall and two-day food record were pooled and divided into 17 food groups, for example, “cereals and cereals products,” “traditional Malaysian kuih and confectionaries” and “fast food,” as shown in Supplementary Table S1 by two independent nutritionists. Then, conceptually similar foods that share comparable features of both nutritional content and manner of serving were aggregated into groups by their fat, carbohydrate, protein, and energy content per portion eaten. Fruits and vegetables were aggregated by their vitamins and minerals content per portion. Foods items reported by less than 15 subjects were then removed. Using stepwise multiple regression analysis, food items that contributed 90% of between-person variation in energy, fat, protein, carbohydrate, vitamins, and minerals were included in the food list. The food list was then reviewed by a group of nutrition experts for suitability of use in diet–disease relationship study.

### 2.4. Development of Nutrient Database

A semiquantitative FFQ can quantify the nutrient intake based on the users’ response from the food list using a nutrient database. Therefore, the Malaysian Food Composition Table [15], the Singapore Food Database [16], and the United States Department of Agriculture nutrient database [17] were used as references for the TMC-derived food list to in the newly developed FFQ. For mixed dishes and cooked foods, a comprehensive and new nutrient composition database for recipes was developed by interviewing cohort participants and referring to cookbooks. As for commercial food products, nutrient composition data from the nutrition information panel supplied by the manufacturers were used. An averaged nutrient composition was used for those food items, which were merged to be a single item. All nutrient compositions were tabled for energy (kcal) and 14 other nutrients per 100 g of each food item.

Reference portion sizes, measured in grams, for each food item in the nutrient database were the median portion size habitually consumed by all subjects, as reported in their 24-h recall and 2-day food records. These portion sizes in grams were then portrayed in household measures, such as a plate, bowl, cup, glass, and spoons of different sizes in the FFQ. When using the FFQ, users were able to estimate their intake portion size based on the listed reference household measurement for each food item. The FFQ was also pretested for clarity of language, ethnic-specific names of foods, and improvement of layout and design among 20 individuals who had similar socio-demographic characteristics to the TMC project participants. Each individual provided their input verbally through an in-depth interview, and all responses were recorded. Most common responses by more than three individuals were addressed directly by making changes to the FFQ, while the importance of other comments was examined by the research team. Whenever there was a contradiction in the comments, the research team selected the choice made by the majority of pretest study participants.

### 2.5. Validation of Food Frequency Questionnaire (FFQ)

In Phase 2, the relative validity of the FFQ was assessed by comparing food intake using FFQ against three datasets of the 24-h dietary recall, using a multiple pass approach, taken once a week for three consecutive weeks, as the reference. During the fourth week, the FFQ was used to measure dietary intake. The subjects were invited to participate for screening at the selected center of each location (first meeting and final meeting for administration of FFQ). The 24-h dietary recall for the 2nd and 3rd weeks were conducted either through a face-to-face interview at the center, a home visit, or a phone interview. All three datasets of the 24-h dietary recall were conducted on different days of the week, and at least one weekend was included.

### 2.6. Nutrient Intake Analysis

Nutrient intake was analyzed using a computer-based comprehensive dietary assessment tool, known as Nutritionist Pro^TM^ (Axxya Systems, Redmond, WA, USA), with the nutrient composition collated from several databases, including the Nutrient Composition of Malaysian Food (MYFCD), USDA Standard Reference Database, Singapore Food Composition Database, Atlas of Food Exchanges & Portion Sizes [18], Canadian Nutrient File, Food and Nutrient Database for Dietary Studies, and Food and Nutrient Database Intake For Diet. Nutrients analyzed were total energy (kcal/day), protein (grams), fat (grams), carbohydrate (gramss), vitamin A (µg), B1 (mg), B2 (mg), B3 (mg), C (mg), calcium (mg), phosphorus (mg), iron (mg), sodium (mg), and potassium (mg).

### 2.7. Statistical Analysis

Stepwise multiple regressions were performed to develop the food list based on the between-person variance in the intake of specific nutrients. Energy intake, fat, protein, carbohydrate, vitamins, and minerals were treated as dependent variables, while the food items were set as the independent variables. Food items with up to a 0.90 cumulative square of the multiple correlation coefficients (R^2^) were selected. In the validation study phase, median was used instead of mean ± standard deviation, due to violation of normality assumption. Spearman correlation was used to estimate the relative validity of FFQ, as compared with the average of the three-day dietary recall. Cutoff for weak, moderate, strong, and very strong correlation coefficients were <0.3, 0.3 to 0.39, 0.4 to 0.69, and ≥0.7, respectively. Meanwhile, intraclass correlation coefficient (ICC) measured reliability coefficients between the two methods for continuous nutrient intakes data. The ICC is calculated as a ratio ICC = (variance of interest)/(total variance) = (variance of interest)/(variance of interest + unwanted variance). If the unwanted variance is equal to or larger than the variance of interest (for example, the variance between subjects), the reliability of the method is evidently poor, and the ICC has a value of below 0.5. On the other hand, ICC values above 0.8 or 0.9 indicated as a good or excellent reliability and less within-person variation. In addition, the Bland–Altman plot was used to assess the agreement between total energy intakes derived using FFQ and 24-h dietary recall (24HDR). Bland–Altman plots were constructed using the difference in energy intake between the two methods (Energy intake FFQ—Energy intake 24HDR) against the mean intake of the two measures ((FFQ + 24HDR)/2). Mean difference in the Bland–Altman indicated whether one method tends to overestimate or underestimate, and the limits of agreement (Mean ± 1.96 SD) were used to portray how well the two dietary intake assessment methods agree [19].

Besides that, subjects were grouped into quartiles for each food groups to test the agreement in ranking participants based on their food consumption as estimated from both methods. The proportions of participants classified into the same, same and adjacent, as well as opposite quartiles for both methods were estimated. The degree of reliability coefficients was evaluated by the weighted Kappa coefficient for categorical data. Cohen’s kappa was performed according to the following formula, where Pr(*a*) represents the actual observed agreement, and Pr(*e*) represents chance agreement:

$$k=\frac{\mathrm{Pr}\left(a\right)-\mathrm{Pr}\left(e\right)}{1-\mathrm{Pr}\left(e\right)}$$

Moderate-to-good agreement was indicated by the weighted Kappa coefficient of >0.40 and acceptable agreement at between 0.20 and 0.39. All analyses were performed using IBM SPSS for Windows version 22 (IBM Corp, Armonk, NW, USA). The significance level was set at 0.05 in all analyses.

## 3. Results

### 3.1. The Characteristics of Study Population

A total of 803 subjects (329 men and 474 women) were included in the development phase, based on the complete dietary data available, and 64 subjects (25 men and 39 women) were included in the validation phase of this study. The demographic characteristics of the subjects are shown in Table 1. The majority were in the 40-to-59-years age group, were Malays, had normal body weight or were overweight, and had at least a secondary education level. Both groups of subjects were recruited from rural and urban area, with similar profiles of place of residence and socioeconomic status.

### 3.2. Energy Intake

Average daily energy intake in the development phase was 1651 ± 389 kcal, as shown in Table 2. The main source of energy was from carbohydrates (51.2%), followed by fat (31.1%) and protein (18.1%). We identified 763 different food items and mixed dishes (Table 3). Grouping of conceptually similar food items resulted in a shorter food list with 161 food items. Stepwise multiple regressions analysis showed that only 152 food items explained 90% of the variance for energy, fat, protein, carbohydrate, vitamins, and minerals intake. An additional 51 food items were included for their importance in diet–disease relationship studies. The final list of food items (n = 203) included in this FFQ is shown in Supplementary Table S1. As for the frequency response format, open-ended response scales (numbers of units taken at a time: “per day,” “week,” and “month”) were chosen, as they reflect the precision with which users can realistically describe their usual intake for the past one year. 

### 3.3. Comparison between Food Frequency Questionnaire and 24-h Diet Recall

As shown in Table 4, the FFQ had a higher estimated median intake of all nutrients (energy, carbohydrate, protein, fat, calcium, phosphorus, iron, potassium, vitamin A, vitamin B2, and vitamin C), except for sodium, compared to the intake, as assessed by repetitive 24-h dietary recalls. Spearman correlation for energy and nutrient intake between both methods showed moderate relative validity for most nutrients. Carbohydrates intake had the highest correlation coefficient (0.45). Moderate correlation coefficients (0.29 to 0.45) were observed for energy (0.36), protein (0.32), fat (0.31), calcium (0.31), phosphorus (0.30), iron (0.42), potassium (0.32), and vitamin A (0.30). In contrast, sodium (0.29) and vitamin C (0.24) showed weak correlations. In addition, intraclass correlation coefficient (ICC) values, which measured the level of agreement between the two methods, also showed a moderate degree of agreement (0.3 to 0.6) for energy intake (0.53), carbohydrates (0.59), protein (0.52), potassium (0.52), vitamin A (0.50) phosphorus (0.48), fat (0.44), and sodium (0.48). Both FFQ and 24-h dietary recall reported poor agreement (0.0 to 0.3) for calcium (−0.01).

**Figure 2:**
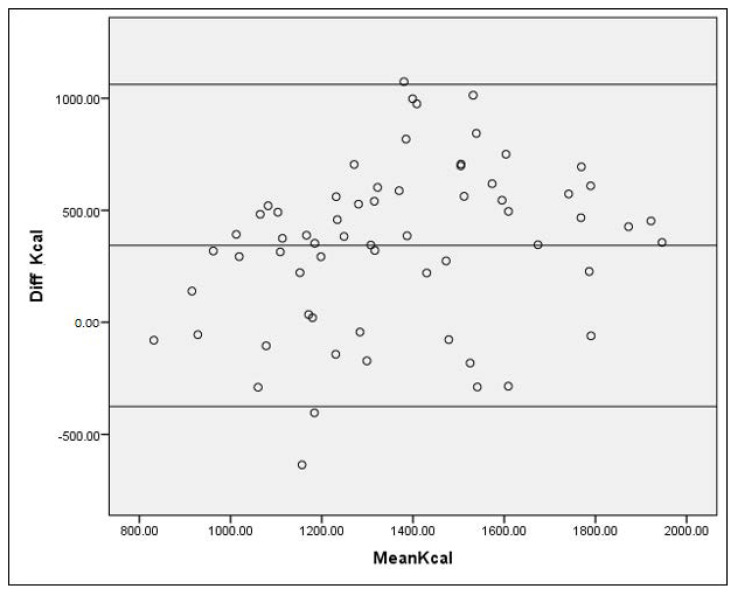
Bland–Altman plot of total energy intake. Differences in the daily intake of total energy estimated with 24-h dietary recalls and a FFQ plotted against the mean daily intake estimated by the two methods (n = 64). Mean difference and 95% limits of agreement (1.96 × SD of mean difference) are included.

The Bland–Altman plot for energy in Figure 2 shows a reasonable level of agreement between the two methods, although there were very few individuals who fell outside the limit of agreement.

Accurate classification of the subjects was possible when their energy or nutrient intakes were ranked into the same or an adjacent quartile by both the methods, as presented in Table 4. The scatterplots of nutrient intakes comparison between FFQ and 24-h dietary recall are shown in Supplementary Figure S1. The proportion of participants classified within the same and adjacent quartile ranged from 29.1% for potassium to 50.5% for vitamin B1. Classification into opposite quartiles varied from 9.7% (energy) to 29.1% (vitamin A). A moderate-to-good agreement in ranking the participants, according to their intake between methods (weighted Kappa > 0.40) was shown for energy (0.6), carbohydrate (0.6), protein (0.5), total fat (0.5), calcium (0.5), sodium (0.5), potassium (0.4), and vitamin B2 (0.5). Furthermore, phosphorus (0.3), iron (0.2), vitamin A (0.2), vitamin B1 (0.3), vitamin B3 (0.3), and vitamin C (0.3) had acceptable agreement (Kappa 0.2 to 0.39) [20].

## 4. Discussion

We have successfully developed a validated 203-item FFQ based on the dietary intake of a considerably large sample of participants from TMC. Overall, the characteristics of the selected subjects were comparable with the original cohort profile, indicating a good representation of the Malaysian Cohort samples [2]. Compared to another national study i.e., the Malaysian Adults Nutrition Survey (MANS) study, [21] the dietary intake reported here is somewhat comparable, except for a higher intake of non-energy-yielding nutrients, such as calcium, iron, sodium, vitamin A, and vitamin C. The MANS study, which utilized only a single 24-h recall, might have introduced underreporting of nutrients, especially on energy, micronutrients, and minerals intake, which are best measured using repetitive recalls or FFQ.

As for the frequency response format, open-ended response scales (numbers of units taken at a time: “per day,” “per week’” and “per month”) were chosen, as they reflect the precision with which users can realistically describe their usual intake [22]. Reference portion sizes measured in grams for each food item in the nutrient database were median portion sizes habitually consumed by all subjects, as reported in their 24-h recall and 2-day food records. We used this approach, as the fitting for portion sizes in an FFQ were previously shown to improve the quantitative assessment of food and nutrient intake [23]. These portion sizes in grams were then portrayed in household measures, such as a plate, bowl, cup, glass, and spoons of different sizes in the FFQ. When using the FFQ, users will be able to estimate their intake portion size based on the listed reference household measurement for each food item.

The strengths of this study include a comprehensive food items list derived from a national-level survey and the development of a dedicated nutrient database using local and adopted values from various food composition tables. It is always a challenge to obtain the nutrient composition of mixed dishes, which are commonly consumed in Malaysia. Recipes vary from different ethnicity and location; therefore, an average was adopted in our nutrient databases to allow for generalization. The number of food items in a FFQ is a pivotal determinant of data accuracy and feasibility of the FFQ. Many semiquantitative FFQs have between 100 and 200 items. According to Cade et al., when using a lengthy food list, users might be influenced to choose more food items, while a shorter food list will restrict their choices [14]. This will lead to incorrect estimates of intakes and will result in over-reporting or underreporting of energy intake. Our FFQ has 203 food items, which we consider to be representative of our multi-ethnic population that can also best provide an accurate picture of dietary habits without becoming a burden to the study participants.

Malaysia has an equatorial climate with uniformly high temperatures, high humidity, relatively light winds, and abundant rainfall throughout the year, common characteristics of a tropical country. It has two monsoons, and there is some seasonal variation on certain fruits and vegetables. However, availability of seasonal foods is not a problem in Malaysia, where fruits and vegetables are highly imported from other countries to meet demand. Therefore, seasonal fruits and vegetables are also included in our FFQ with no corrections of intake being made for seasonal variation [24]. In Malaysia, alcohol intake has a negative religious and social perception and, therefore, is not commonly reported by the participants of this FFQ development phase. Thus, common alcoholic beverages were added to the FFQ due to the interest of identifying their relationship with the noncommunicable diseases in Malaysia. In contrast, food preferences for protein-based foods were mostly related to religion, and it was evident that pork intake was not reported by Muslims, while beef was not reported by the Hindus and some Buddhists. Being culturally sensitive, we, therefore, created a separate section for them so that those not consuming specific meat items would be able to skip the section.

We have also evaluated the relative validity of our FFQ among 64 participants of TMC project. Our results have demonstrated moderate relative validity for almost all macro- and micronutrients. Besides that, the Bland–Altman plot, an alternative way to indicate agreement, showed an acceptable level of agreement between the two methods. Findings of the current study have shown relative validity between the correlation coefficient range of 0.24 to 0.46. This is similar to another FFQ validation study against 24-h dietary recall among 161 adults within the age group of 18 to 80 years old who participated in a German Health Examination Survey, which reported a Spearman correlation coefficient in the range of 0.15 to 0.8, with most values exceeding 0.3 [25]. We also observed a moderate-to-fair rate of agreement based on Kappa statistics between FFQ and 24-h dietary recall, especially for energy and macronutrients. This might be due to the appropriate time interval, i.e., approximately 30 days between the two dietary assessment methods. Duration between FFQ and 24-h dietary recall should not be too close or far apart to prevent seasonal or daily changes in dietary habits. Marques-Vidal and colleagues have proposed 15 days to one year as the appropriate time interval between two dietary assessment methods. In addition, their study determining validity of FFQ against 24-h dietary record among 40 adults employed an interval of one month between the two methods, which is similar to the duration of current study [26]. Furthermore, to minimize bias and obtain a dietary information which is more accurate, we have conducted our dietary recalls on unscheduled days.

The results from validation phase of the current FFQ also showed that estimation of sodium and vitamin C needs some improvement. Since sodium is mainly hidden in processed and cooked foods, the food composition database and the calculation of standardized recipes will be revisited as a mitigation plan to improve the validity of this FFQ. Recipes also vary from different ethnicity and location; therefore, an average was adopted in our nutrient databases to allow for generalization. As for vitamin C, the list of fruits in the FFQ would further be reduced by removing items that do not appear in any 24-h diet recall to avoid overestimation of intake. In addition, the most crucial part for the evaluation of FFQ is the selection of the appropriate reference method [27]. A review by Cade and colleagues have found that almost 75% studies on FFQ validation used 24-h dietary recall as the gold standard, as has been adopted in the present study [28]. The FFQ and 24-h dietary recall have distinct differences and their own independent source of errors. The 24-h dietary recalls rely on short-term memory, whilst FFQ on long-term memory. Thus, in the validation phase of the present study, three repetitive 24-h recalls over a three-week period was conducted prior to administration of the FFQ. It should be noted that the dietary recall method requires a trained interviewer who asks open-ended questions, while FFQ consists of close-ended, self-administered questions. Both systematic and random errors exist in every dietary assessment method; thus, there is no universally acceptable gold standard tool to be used as comparison with FFQ. For any validation study, errors for the dietary tools chosen should not be correlated with one another [29]. Since errors of both the methods are independent of each other, the 24-h dietary recall is suitable to be used as the comparison with FFQ [25].

There were two main limitations associated with this newly developed FFQ. First, our FFQ was developed to be interviewer-administered to avoid misreporting by participants. This might incur a higher cost, as a suitable number of interviewers would be needed in a large cohort study, such as in TMC project. They will also need to be trained periodically to minimize inter-interviewer bias. A multi-ethnic population, as in Malaysia, would also have varied languages and accents. Therefore, the FFQ should be translated into various main languages in Malaysia in future. Secondly, the use of FFQ with a population which mainly consumes mixed dishes would be lengthy. This could not be avoided and will increase the time spent for the interview. The estimation of intake by respondents would also become difficult and should be used with caution among elderly.

Due to the limitation of the use of 24-h dietary recall, which relies solely on participants’ memory as the gold standard for FFQ validation, another method could be considered in future studies. A more accurate method for reference is by using biochemical measurements (biomarkers) of nutrients in blood or other tissues, such as urinary nitrogen [30]. Although this approach will have a higher degree of accuracy, biomarkers related experiments are expensive, time consuming, complex, and nutrient-specific, where they are only able to validate one nutrient at a time. Furthermore, better estimation or correlation can be obtained when dietary recall is collected for at least three days, as has been conducted in the present study.

On a positive note, interviewer-administered FFQ is more preferred than self-administered FFQ in obtaining quality data if cost is not an issue [31]. This would also help older participants with low literacy. TMC project also has developed a mechanism to conduct quality control by listening to recordings of interviews, which would not be possible if questionnaires were self-administered. In addition, due to a comprehensive list of food items in our FFQ, a minimum of 20 min was needed to administer the FFQ for each participant. However, the majority of participants who consented to be part of TMC project provided full cooperation. In order to ease the burden on participants, the layout and design of FFQ were arranged so that participants could screen which foods were consumed habitually by them. Further questions on frequency of intake and serving size focused on the selected common foods only. We shall be taking this study further to evaluate the feasibility of an online interview-based system and to determine the cost-effectiveness of this approach.

## 5. Conclusions

We have successfully developed and validated a FFQ based on a list of 203 food items for estimating food intake of a multi-ethnic Malaysian population for TMC project. This culturally specific FFQ is pivotal in estimating dietary exposure in a large cohort study and in identifying the role of diet in determining the risk for noncommunicable diseases in Malaysia. Future studies will address the feasibility and cost-effectiveness of an online interview-based system of this newly developed semiquantitative FFQ.

## Figures and Tables

**Table 1:** Demographic characteristics of subjects from development (n = 803) and validation phase (n = 64).

Demographics	Categories	Development			Validation		
		Men	Women	Total	Men	Women	Total
		(n = 329)	(n = 474)	(n = 803)	(n = 25)	(n = 39)	(n = 64)
**Age, mean (SD) years**		50.5 (7.9)	48.3 (7.4)	49.2 (7.7)	58.6 (6.6)	54.8 (6.0)	56.3 (6.5)
**Age group, n (%) (years)**	40 to 49	147 (44.7)	262 (55.3)	409 (50.9)	3 (12.0)	8 (20.5)	11 (17.2)
	50 to 59	134 (40.7)	175 (36.9)	309 (38.5)	11 (44.0)	22 (56.4)	33 (51.6)
	60 and above	48 (14.6)	37 (7.8)	85 (10.6)	11 (44.0)	9 (23.1)	20 (31.3)
**BMI, mean (SD) kg/m^2^**		25.5 (4.0)	25.8 (4.9)	25.5 (4.0)	25.7 (3.7)	26.0 (4.3)	25.9 (4.0)
**BMI classification, n (%)**	Underweight	13 (4.0)	20 (4.2)	13 (4.0)	1 (4.0)	1 (2.6)	2 (3.1)
	Normal weight	139 (42.2)	197 (41.6)	139 (42.2)	9 (36.0)	16 (41.0)	25 (39.1)
	Overweight	139 (42.2)	184 (38.8)	139 (42.2)	12 (48.0)	14 (35.9)	26 (40.6)
	Obese	38 (11.6)	73 (15.4)	38 (11.6)	3 (12.0)	8 (20.5)	11 (17.2)
**Ethnicity, n (%)**	Malay	178 (54.1)	176 (37.1)	178 (54.1)	17(68.0)	18 (46.2)	35 (54.7)
	Chinese	79 (24.0)	180 (38.0)	79 (24.0)	7 (28.0)	18 (46.2)	25 (39.1)
	Indian	38 (11.6)	84 (17.7)	38 (11.6)	1 (4.0)	3 (7.7)	4 (6.2)
	Others	34 (10.3)	34 (7.2)	34 (10.3)			
**Place of residence, n (%)**	Rural	158 (48.0)	231 (48.7)	158 (48.0)	17 (68.0)	16 (41.0)	33 (51.6)
	Urban	171 (52.0)	243 (51.3)	171 (52.0)	8 (32.0)	23 (59.0)	31 (48.4)
**Education level, n (%)**	No schooling	5 (1.5)	10 (2.1)	15 (1.9)	1 (4.0)	2 (5.1)	3 (4.7)
	Primary	87 (26.4)	127 (26.8)	214 (26.7)	16 (64.0)	14 (35.9)	30 (46.9)
	Secondary	140 (42.6)	213 (44.9)	353 (44.0)	6 (24.0)	15 (38.5)	21 (32.8)
	Tertiary	97 (29.5)	124 (26.2)	221 (27.5)	2 (24.0)	8 (20.5)	10 (15.6)
**Household income (USD), n (%)**	<USD 241				0 (0)	2 (5.10)	2 (3.10)
	USD 241–481.77				7 (28.00)	12 (30.80)	19 (29.70)
	USD 482.01–722.77				11 (44.00)	5 (12.80)	16 (25.00)
	USD 723.01–963.78				2 (8.00)	5 (12.80)	7 (10.90)
	USD 964.02–1204.78				4 (16.00)	3 (7.70)	7 (10.90)
	USD 1205.03–1686.79				0 (0)	4 (10.30)	4 (6.30)
	USD 1687.04–2409.81				1 (4.00)	5 (12.80)	6 (9.40)
	>USD 2410.05				0 (0)	3 (7.70)	3 (4.70)

**Table 2:** Nutrient intake of the subjects from the development phase (n = 803).

Parameters	Men	Women	Total	*p*-Value ^1^
	(n = 329)	(n = 474)	(n = 803)	
Energy (kcal/day)	1741 (437)	1589 (339)	1651 (389)	<0.01
Carbohydrates (g/day)	224.9 (68.2)	199.9 (52.7)	210.2 (60.8)	<0.01
% from energy	52.0 (10.1)	50.6 (10.0)	51.2 (10.1)	
Protein (g)	77.2 (36.6)	72.8 (25.9)	74.6 (30.8)	0.06
% from energy	17.7 (6.7)	18.3 (5.1)	18.1 (5.8)	
Total fat (g)	60.8 (29.1)	55.6 (20.9)	57.7 (24.8)	0.01
% from energy	31.1 (11.8)	31.1 (7.7)	31.1 (9.6)	
Calcium (mg)	484.9 (235.9)	474.7 (266.5)	478.9 (254.3)	0.58
Phosphorus (mg)	1006.1 (535.5)	919.3 (421.6)	954.9 (473.2)	0.14
Iron (mg)	15.6 (8.7)	14.3 (6.2)	14.9 (7.3)	0.02
Sodium (mg)	3217.3 (2063.2)	3132.3 (2180.3)	3167.2 (2132.2)	0.58
Potassium (mg)	1512.3 (969.8)	1400.1 (650.0)	1446.1 (798.1)	0.07
Zinc (mg)	5.2 (4.3)	5.5 (5.0)	5.4 (4.72)	0.4
Vitamin A (µg)	722.0 (883.3)	597.9 (1715.2)	648.7 (1434.5)	0.23
Vitamin B1 (mg)	0.8 (0.5)	0.8 ± 0.4	0.8 (0.4)	0.22
Vitamin B2 (mg)	1.3 (0.9)	1.2 (0.6)	1.2 (0.7)	0.04
Vitamin B3 (mg)	9.2 (5.4)	8.7 (4.8)	8.9 (5.1)	0.11
Vitamin C (mg)	115.9 (342.0)	156.1 (252.2)	139.6 (292.7)	0.47

^1^*p*-value < 0.05 (2-tailed). Values are presented in mean (SD).

**Table 3:** Food categories of the semiquantitative food frequency questionnaire derived from 803 participants.

Food Categories	Total Food Items and Mixed Dishes ^1^	Grouping of Food Items ^2^	Contribution of 90% ^3^	Inclusion of Foods ^4^	Final Food Items ^5^
Cereal	104	26	26	7	33
Meat	68	16	15	5	20
Fish and shellfish	143	15	15	5	20
Egg	10	3	3	0	3
Vegetables	128	20	19	2	21
Tuber and starch	11	4	3	1	4
Soy products	17	3	3	2	5
Beans and legumes	13	3	3	1	4
Fruits	30	18	17	3	20
Milk and milk products	17	6	4	5	9
Fast foods	16	7	6	7	13
Beverages	71	16	16	2	18
Alcoholic beverages	0	0	0	3	3
Traditional snacks and confectionaries	104	9	9	4	13
Condiments and gravies	21	7	6	0	6
Spreads	8	6	4	3	7
Sweetener	2	2	2	2	4
Total	763	161	151	52	203

^1^ Total number of exact food items and mixed dishes derived from all participants (n = 803) from development phase, excluding duplicate food items. ^2^ Number of food items and mixed dishes after aggregation based on similar nutritional content, manner of serving, excluding food items reported by less than 15 subjects. ^3^ Food items that contributed 90% of between-person variation in energy, fat, protein, carbohydrate, vitamins, and minerals using stepwise multiple regression analysis. ^4^ Inclusion of foods after being reviewed by experts based on their importance in diet–disease relationship study. ^5^ Final food items included in the food frequency questionnaire (FFQ).

**Table 4:** Comparison of nutrient intakes and quartiles joint classifications between food frequency questionnaire (FFQ) and 3-day diet recall (3DR) (n = 64).

Nutrient (Unit)	FFQ	3DR	*p*-Value ^1^	S.C.C., rs	I.C.C.	S.Q. (%)	S.A.Q. (%)	O.Q. (%)	Q.W.K.
	Median (I.Q.R.)	Median (I.Q.R.)							
Energy (kcal)	1495 (1148–1615)	1152 (959–1385)	<0.001 **	.364 **	0.53 **	40.6	70.3	7.8	0.25 (0.01 to 0.49)
Carbohydrates (g)	201 (177.2–246.5)	167.9 (131.0–190.5)	<0.001 **	.456 **	0.59 **	34.4	68.8	3.1	0.45 (0.24 to 0.66)
Protein (g)	56.9 (47.7–71.1)	48.4 (38.8–62.7)	0.003 **	.329 **	0.52 **	42.2	81.3	4.7	0.30 (0.09 to 0.51)
Fat (g)	50.2 (39.7–59.3)	34.9 (26.6–44.5)	<0.001 **	.316 *	0.44 *	42.2	70.3	3.1	0.35 (0.13 to 0.57)
Calcium (mg)	453.2 (323.2–545.7)	363.4 (274.8–474.8)	0.010 *	.312 *	−0.01	28.1	73.4	4.7	0.30 (0.09 to 0.51)
Phosphorus (mg)	834.5 (697.3–1004.8)	631 (508.2–829.0)	<0.001 **	.301 *	0.48 **	34.4	71.9	6.3	0.28 (0.05 to 0.50)
Iron (mg)	12.32 (8.6–15.3)	10.2 (7.5–13.9)	0.1	0.425 **	0.38 *	42.2	79.7	6.3	0.40 (0.18 to 0.62)
Sodium (mg)	1520.2 (1230.7–1968.3)	1618.9 (1240.0–2307.0)	0.07	0.291 *	0.48 **	28.1	75	6.3	0.29 (0.07 to 0.51)
Potassium (mg)	1325.5 (988.1–1783.3)	1099 (840.8–1406.3)	0.002 **	0.326 **	0.523 **	37.5	76.6	4.7	0.38 (0.16 to 0.59)
Vitamin A (µg)	665.5 (349.8–617.0)	495.2 (349.8–617.0)	<0.001 **	0.303 *	0.50 **	29.7	71.9	4.7	0.29 (0.07 to 0.50)
Vitamin C (mg)	92.1 (53.8–145.7)	57.1 (33.7–117.8)	0.01 *	0.239	0.40 *	29.7	62.5	4.7	0.18 (−0.05 to 0.40)

S.C.C. indicates Spearman correlation coefficient; I.C.C. indicates interclass correlation coefficient; Q.W.K. indicates Quadratic weighted Kappa; I.Q.R. indicates interquartile range, S.Q. indicates same quartile (%), S.A.Q. indicates same and adjacent quartile (%), O.Q. indicates opposite quartile and 95% CI indicates 95% confidence interval ^1^ Wilcoxon Signed Rank Test. * *p*-value < 0.05. ** *p*-value < 0.01.

## Data Availability

The datasets used and/or analyzed during the current study are available from the corresponding author on reasonable request.

## Supplementary Materials

The following are available online at https://www.mdpi.com/article/10.3390/nu13041163/s1, Figure S1: Comparison of nutrient intakes between FFQ and 3DR, Table S1: Complete food group and list included in the semi-quantitative FFQ.

## References

[1] Institute for Public Health (2015). National Health and Morbidity Survey 2015 (NHMS 2015). Vol. II: Non-Communicable Diseases, Risk Factors & Other Health Problems.

[2] Jamal R., Syed Zakaria S.Z., Kamaruddin M.A., Abd Jalal N., Ismail N., Mohd Kamil N., Abdullah N., Baharudin N., Hussin N.H., Othman H. (2015). Cohort Profile: The Malaysian Cohort (TMC) project: A prospective study of non-communicable diseases in a multi-ethnic population. Int. J. Epidemiol..

[3] Sulaiman S., Shahril M.R., Shaharudin S.H., Md Isa N., Syed Hussain S.N.A. (2008). Semi-Quantitative Food Frequency Questionnaire for Assessment of Energy, Total Fat, Fatty Acids, and Vitamin A, C and E Intake among Malaysian Women: Comparison with Three Days 24-Hour Diet Recalls. J. Sains Kesihat. Malays..

[4] Satija A., Yu E., Willett W.C., Hu F.B. (2015). Understanding Nutritional Epidemiology and Its Role in Policy. Adv. Nutr..

[5] Bao Y., Bertoia M.L., Lenart E.B., Stampfer M.J., Willett W.C., Speizer F.E., Chavarro J.E. (2016). Origin, Methods, and Evolution of the Three Nurses’ Health Studies. Am. J. Public Health.

[6] Cade J.E., Burley V.J., Alwan N.A., Hutchinson J., Hancock N., Morris M.A., Threapleton D.E., Greenwood D.C. (2017). Cohort Profile: The UK Women’s Cohort Study (UKWCS). Int. J. Epidemiol..

[7] Gonzalez C.A. (2006). The European Prospective Investigation into Cancer and Nutrition (EPIC). Public Health Nutr..

[8] Dao M.C., Subar A.F., Warthon-Medina M., Cade J.E., Burrows T., Golley R.K., Forouhi N.G., Pearce M., Holmes B.A. (2019). Dietary assessment toolkits: An overview. Public Health Nutr..

[9] Khairunnisa M. (2018). The Development of Food Frequency Questionnaire and Assessment of 15-Year-Old Malaysian Adolescent Dietary Intake. Ph.D. Thesis.

[10] Fatihah F., Ng B., Hazwanie H., Norimah A., Nik Shanita S., Ruzita A., Poh B. (2015). Development and validation of a food frequency questionnaire for dietary intake assessment among multi-ethic primary school-aged children. Singap. Med. J..

[11] Zaleha M.I., Khadijah S., Bukhary I.B.N., Khor G.L., Zaleha A.M., Haslinda H., Hana Y.N.S., Hasanain Faisal G. (2015). Development and Validation of a Food Frequency Questionnaire for Vitamin D intake among Urban Pregnant Women in Malaysia. Malays. J. Nutr..

[12] Shahar S., Hui Lin C., Haron H. (2014). Development and Validation of Food Frequency Questionnaire (FFQ) for Estimation of the Dietary Polyphenol Intake among Elderly Individuals in Klang Valley. J. Sains Kesihat. Malays..

[13] Lee L.K., Shahar S., Yusoff N.A.M., Chin A.-V. (2013). Validation of a food frequency questionnaire in assessing the omega-3 polyunsaturated fatty acids intake for Malays and Chinese elderly in Malaysia. Sains Malays..

[14] Cade J., Thompson R., Burley V., Warm D. (2002). Development, validation and utilisation of food-frequency questionnaires—A review. Public Health Nutr..

[15] Tee E.S., Noor M.I., Azudin M.N., Idris K. (1997). Nutrient Composition of Malaysian Foods.

[16] Energy and Nutrient Composition of Food. https://focos.hpb.gov.sg/eservices/ENCF/.

[17] United States Department of Agriculture: Agricultural Research Service. https://ndb.nal.usda.gov/ndb/.

[18] Abdul Manaf Z., Shahar S., Safii N.S., Haron H. (2015). Atlas of Food Exchanges & Portion Sizes.

[19] Bland J.M., Altman D.G. (1986). Statistical methods for assessing agreement between two methods of clinical measurement. Lancet.

[20] Lombard M.J., Steyn N.P., Charlton K.E., Senekal M. (2015). Application and interpretation of multiple statistical tests to evaluate validity of dietary intake assessment methods. Nutr. J..

[21] Norimah A.K., Safiah M., Jamal K., Haslinda S., Zuhaida H., Rohida S., Fatimah S., Norazlin S., Poh B.K., Kandiah M. (2008). Food Consumption Patterns: Findings from the Malaysian Adult Nutrition Survey (MANS). Malays. J. Nutr..

[22] Jayawardena R., Swaminathan S., Byrne N.M., Soares M.J., Katulanda P., Hills A.P. (2012). Development of a food frequency questionnaire for Sri Lankan adults. Nutr. J..

[23] Nöthlings U., Hoffmann K., Bergmann M.M., Boeing H. (2007). Fitting Portion Sizes in a Self-Administered Food Frequency Questionnaire. J. Nutr..

[24] Abdullah N.-F., Teo P., Foo L. (2016). Ethnic Differences in the Food Intake Patterns and Its Associated Factors of Adolescents in Kelantan, Malaysia. Nutrients.

[25] Haftenberger M., Heuer T., Heidemann C., Kube F., Krems C., Mensink G.B. (2010). Relative validation of a food frequency questionnaire for national health and nutrition monitoring. Nutr. J..

[26] Marques-Vidal P., Ross A., Wynn E., Rezzi S., Paccaud F., Decarli B. (2011). Reproducibility and relative validity of a food-frequency questionnaire for French-speaking Swiss adults. Food Nutr. Res..

[27] Liu X., Wang X., Lin S., Song Q., Lao X., Yu I.T.-S. (2015). Reproducibility and Validity of a Food Frequency Questionnaire for Assessing Dietary Consumption via the Dietary Pattern Method in a Chinese Rural Population. PLoS ONE.

[28] Cade J.E., Burley V.J., Warm D.L., Thompson R.L., Margetts B.M. (2004). Food-frequency questionnaires: A review of their design, validation and utilisation. Nutr. Res. Rev..

[29] Streppel M.T., de Vries J.H., Meijboom S., Beekman M., de Craen A.J., Slagboom P.E., Feskens E.J. (2013). Relative validity of the food frequency questionnaire used to assess dietary intake in the Leiden Longevity Study. Nutr. J..

[30] Picó C., Serra F., Rodríguez A.M., Keijer J., Palou A. (2019). Biomarkers of Nutrition and Health: New Tools for New Approaches. Nutrients.

[31] Kowalkowska J., Wadolowska L., Czarnocinska J., Czlapka-Matyasik M., Galinski G., Jezewska-Zychowicz M., Bronkowska M., Dlugosz A., Loboda D., Wyka J. (2018). Reproducibility of a Questionnaire for Dietary Habits, Lifestyle and Nutrition Knowledge Assessment (KomPAN) in Polish Adolescents and Adults. Nutrients.

