# Supplementary material for: Development and Relative Validity of a Semiquantitative Food Frequency Questionnaire to Estimate Dietary Intake among a Multi-Ethnic Population in the Malaysian Cohort Project
Source: Nutrients. 2021 Apr 1;13(4):1163. doi: 10.3390/nu13041163 (PMC8066188; doi:10.3390/nu13041163)
Supplement: Supplementary file 1 [file nutrients-13-01163-s001.pdf]

nutrients

MDPI

**Supplementary Table S1: Complete food group and list included in the semi-quantitative FFQ.**

**A. Cereals & cereals products**

1. White rice
2. Basmati rice
3. Brown rice
4. Fried rice
5. Rice/ 'Nasi Dagang'
6. Rice/ 'Nasi Arab'/ 'Nasi Briyani'/ 'Nasi Jagung'/ 'Nasi Minyak'/ 'Nasi Tomato'
7. Rice cooked with coconut milk/ 'Nasi Lemak'
8. Chicken Rice
9. Rice/ 'Nasi Kerabu'
10. Plain Porridge
11. Porridge (e.g.: chicken porridge/ fish porridge/ meat porridge/ *lambuk* porridge)
12. Fried Noodles ('mee/ mee-hoon/ kueh-teow/ wantan mee')
13. Noodles cooked with gravy (e.g.: 'hailam/ kicap/ kolok/ sizzling')
14. Noodles cooked with coconut milk gravy (e.g.: 'bandung/ kari/ rebus')
15. Noodles with soup (e.g.: 'mee/ mee-hoon/ kueh-teow/ wantan mee soup')
16. Penang Noodle 'Laksa'
17. Noodle 'Laksa/laksam' with coconut milk gravy
18. 'Roti Canai'
19. Chapatti
20. 'Tosai'
21. Breakfast cereals
22. Oats
23. White bread
24. Wholemeal bread
25. Whole grain bread
26. Sweet bun
27. Sandwich
28. Plain Crackers
29. Wholemeal cracker
30. Sweet biscuits (e.g.: sugar/ Marie/ chocolate)
31. Biscuits with cream (e.g.: chocolate cream/ peanut butter cream/ cheese cream)
32. Fried dimsum/ wantan

**D. Eggs**

74. Boiled egg
75. Fried egg
76. Salted egg

**E. Vegetables**

77. Fried green leafy vegetables
78. Green leafy vegetables soup
79. Green leafy vegetables cooked with coconut milk gravy (e.g.: curry/ 'lemak')
80. Green leafy vegetables cooked by steamed/ boiled
81. Fried cruciferous vegetables
82. Cruciferous vegetables soup
83 Cruciferous vegetables cooked with coconut milk gravy (e.g.: curry/ 'lemak')
84. Cruciferous vegetables cooked by steamed/ boiled
85. Fried bean vegetables
86. Bean vegetables soup
87 Bean vegetables cooked with coconut milk gravy (e.g.: curry/ 'lemak')
88. Bean vegetables cooked by steamed/ boiled
89. Fried starchy vegetables
90. Starchy vegetables soup
91. Starchy vegetables cooked with coconut milk gravy (e.g.: curry/ 'lemak')
92. Starchy vegetables cooked by steamed/ boiled
93. Fried fruit vegetables
94. Fruit vegetables soup
95. Fruit vegetables cooked with coconut milk gravy eg: curry/ 'lemak')
96. Fruit vegetables cooked by steamed/ boiled
97. 'Ulam' (e.g.: cucumber/ tomato/ salad/ 'petai')

**F. Starchy Roots Tubers & Products**

98. Fried Potato
99. Potato cooked with coconut milk (e.g.: curry/ 'kurma/ lemak')

**L. Traditional Malaysian 'Kuih' & Confectionaries**

144. Fried & spicy traditional kuih (e.g.: curry puff/ samosa/ popia rolls/ vadai)
145. Fried & sweet traditional kuih (e.g.: doughnut / 'ham chi peng/ laddoo/ cakoi')
146. Grilled/ Steamed spicy traditional kuih (e.g.: grilled glutinous rice/ 'cara berlauk/ meat pau')
147. Grilled/ Steamed sweet traditional kuih (e.g.: apam/ kuih ang koo/ kaya pau/ kuih talam)
148. Sweet porridge (e.g.: durians porridge/ green beans porridge/ sweet potato porridge)
149. Cakes
150. Pastry (e.g.: pie/ croissant)
151. Jelly/ 'agar-agar'
152. 'Ais kacang/ Cendol'
153. Ice cream
154. Chocolate
155. Sweets
156. 'Kerepek/ keropok/ muruku'

**M. Fastfood**

157. Fried chicken with flour (e.g.: chicken chop)
158. Burger/ hotdog
159. Special Burger (e.g.: burger 'bakar'/ extra cheese/ extra egg)
160. Pizza
161. Spaghetti Bolognese
162. Spaghetti Carbonara
163. Sausage/ Frankfurter
164. Nugget
165. French fries
166. Wedges
167. Mashed potato
168. Coleslaw
169. Creamy mushroom soup

**N. Drink & Beverages**

170. Plain water
171. Tea 'O' (with sugar)
172. Tea 'Tarik' (with milk)
173. 3 in 1 instant tea
174. Coffee 'O' (with sugar)

| | | |
|---|---|---|
| 33. Dimsum/ wantan cooked by steamed/ boiled<br>**B. Meat & Meat product**<br>34. Fried chicken<br>35. Chicken cooked with coconut milk (e.g.: curry/ 'kurma/ lemak/ percik/ rendang')<br>36. Chicken cooked without coconut milk (e.g.: with chilli/ cooked in soy sauce/ with tomato)<br>37. Chicken cooked in soup/ 'asam pedas/ tomyam'<br>38. Roasted/Grilled Chicken<br>39. Chicken satay<br>40. Fried Meat<br>41. Meat cooked with coconut milk (e.g.: curry/ 'kurma/ lemak/ rendang')<br>42. Meat cooked without coconut milk (e.g.: with chilli/ cooked in soy sauce/ with tomato)<br>43. Meat cooked in soup/ 'asam pedas/ tomyam'<br>44. Roasted/Grilled Meat<br>45. Meat Satay<br>46. Fried internal organ<br>47. Internal organ cooked with coconut milk (e.g.: curry/ 'kurma/ lemak/ percik/ rendang')<br>48. Internal organ cooked without coconut milk (e.g.: with chilli/ cooked in soy sauce/ with tomato)<br>49. Internal organ satay<br>50. Fried pork<br>51. Pork cooked without coconut milk (e.g.: with chilli/ cooked in soy sauce/ with tomato)<br>52. Pork cooked in soup/ 'asam pedas/ tomyam'<br>53. Roasted/ grilled pork<br><br>**C. Fish & Fish product**<br>54. Fried fish<br>55. Fish cooked with coconut milk (e.g.: curry/ 'kurma/ lemak/ percik/ rendang')<br>56. Fish cooked without coconut milk (e.g.: with chilli/ cooked in soy sauce/ with tomato)<br>57. Fish cooked in soup/ 'asam pedas/ tomyam' | 100. Potato cooked without coconut milk (e.g.: with chilli/ cooked in soy sauce)<br>101. Potato cooked by steamed/ boiled<br><br>**G. Soys & soy product**<br>102. Fried soy product<br>103. Soy product cooked with coconut oil (e.g.: curry/ 'lemak')<br>104. Soy product cooked without coconut oil milk (e.g.: with chilli/ cooked in soy sauce)<br>105. Soy product cooked by steamed/ boiled<br>106. Soy product grilled<br><br>**H. Nuts**<br>107. Fried nuts<br>108. Nuts cooked by steamed/ boiled<br>109. Nuts gravy<br>110. Sambhar<br><br>**I. Seasonings & Condiments**<br>111. Salted / Sweet soy sauces/ 'sambal kicap'<br>112. Chilli / Tomato sauces<br>113. 'Sambal belacan'/ 'budu'/ 'cencalok'<br>114. Stir fry sauces ('sambal tumis')<br>115. Rasam<br>116. Chutney<br><br>**J. Spread**<br>117. Margarine<br>118. Butter<br>119. Fruits jam<br>120. Kaya jam<br>121. Peanut butter<br>122. Chocolate spread<br>123. Mayonise/ salad dressing<br><br>**K. Fruits**<br>124. Grapes<br>125. Papaya<br>126. Pears<br>127. Dragon fruit<br>128. Jackfruit<br>129. Green/ red apples<br>130. Guava<br>131. Kiwi | 175. Coffee with milk<br>176. 3 in 1 instant coffee<br>177. Malted drink/ chocolate malted drink 'O' (with sugar)<br>178. Malted drink/ chocolate malted drink with milk<br>179. 3 in 1 instant malted drink<br>180. Chinese tea<br>181. Green tea<br>182. Cordial drink<br>183. Carbonated drink/ isotonic drink<br>184. Energy drink<br>185. Soy drink<br>186. Fresh fruits juice (e.g.: apple/ mango/ orange)<br>187. Commercial fruits juice (e.g.: apple/ mango/ orange)<br><br>**O. Food additives**<br>188. Condensed milk<br>189. White sugar<br>190. Honey<br>191. Artificial sweetener<br><br>**P. Milk & milk products**<br>192. Fresh milk<br>193. Skimmed milk powder<br>194. Low fat milk powder<br>195. Full cream milk powder<br>196. Low fat UHT Milk<br>197. Full cream UHT Milk<br>198. Yogurt unflavoured<br>199. Yogurt with flavours<br>200. Cheese<br><br>**Q. Alcoholic beverages**<br>201. Beer/ ale<br>202. Wine/ champagne/ brandy<br>203. Whiskey/ vodka/ 'samsu/ toddy/ tuak/ langkau' |

| | | |
|---|---|---|
| 58. Roasted/grilled fish<br>59. Fried anchovies/ with chilli<br>60. Canned fish with chilli (e.g.: sardines/tuna)<br>61. Fried salted fish<br>62. Fish ball/ fishcakes<br>63. Fried seafood<br>64. Seafood cooked with coconut milk (e.g.: curry/ 'kurma/ lemak')<br>65. Seafood cooked without coconut milk (e.g.: with chilli/ cooked in soy sauce/ with tomato)<br>66. Seafood cooked in soup/ 'asam pedas/ tomyam'<br>67. Roasted/grilled seafood<br>68. Shellfish cooked with coconut milk (e.g.: 'lemak/ rendang')<br>69. Shellfish cooked with chilli<br>70. Shellfish cooked by steamed/ boiled<br>71. Crab cooked with coconut milk (e.g.: 'lemak/ rendang')<br>72. Crab cooked with chilli<br>73. Crab cooked by steamed/ boiled | 132. Tangerines / oranges<br>133. Mango<br>134. Pineapples<br>135. Banana<br>136. Watermelon<br>137. Honeydew/ rock melon<br>138. *Plum*<br>139. Fruit cooked with coconut oil (e.g.: curry/ 'lemak')<br>140. Fruit 'acar' / Fruit 'kerabu'<br>141. Lychee/ Longan<br>142. Dates<br>143. Raisins | |

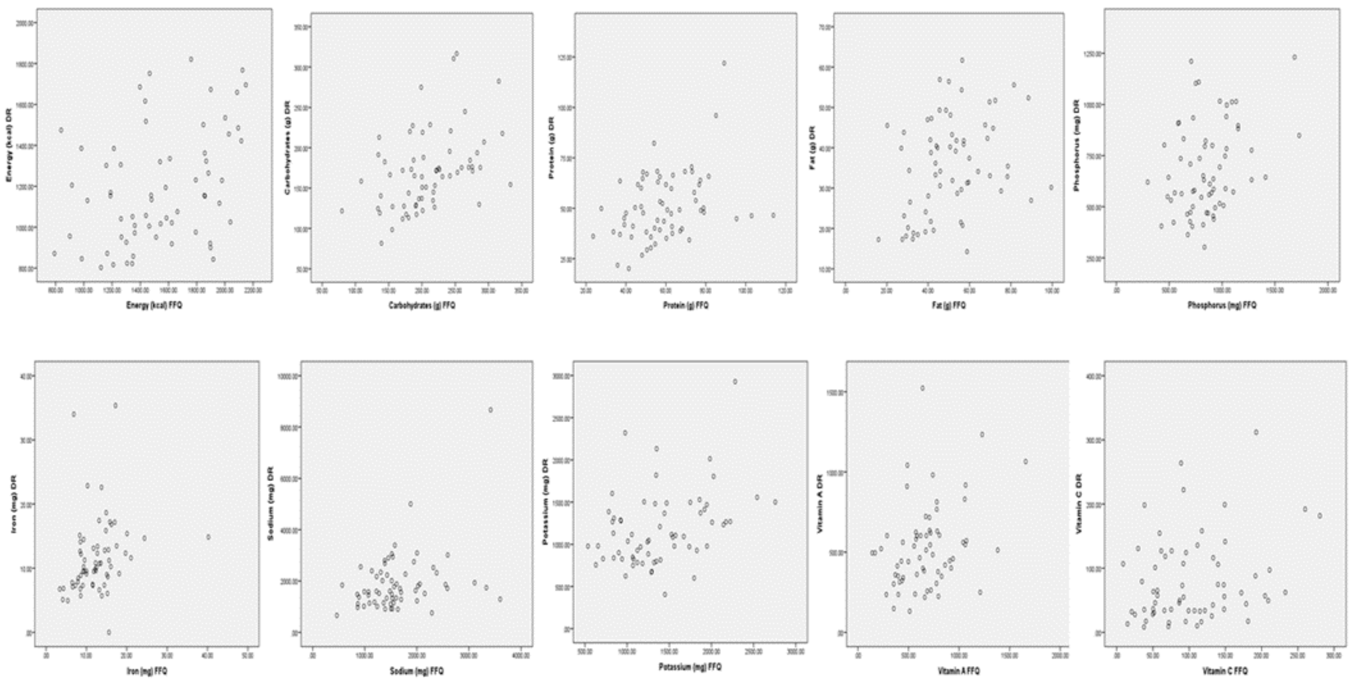


**Supplementary Figure S1:** Comparison of nutrient intakes between FFQ and 3DR
